# Fluoroquinolone-Resistant *Escherichia coli* Infections After Transrectal Biopsy of the Prostate in the Veterans Affairs Healthcare System

**DOI:** 10.20411/pai.v1i2.123

**Published:** 2016-09-20

**Authors:** Elie A. Saade, Nuntra Suwantarat, Trina F. Zabarsky, Brigid Wilson, Curtis J. Donskey

**Affiliations:** 1 Infectious Diseases Division, University Hospitals, Cleveland, Ohio; 2 Case Western Reserve University School of Medicine, Cleveland, Ohio; 3 Infection Control Department, Louis Stokes Cleveland VA Medical Center, Cleveland, Ohio; 4 Geriatric Research Education and Clinical Center, Cleveland VA Medical Center, Cleveland, Ohio

**Keywords:** bacteremia, case-control studies, cohort studies, early detection of cancer, Escherichia coli infections, microbial drug resistance, postoperative complications, prostate, retrospective studies, United States Department of Veterans Affairs, urinary tract infections

## Abstract

**Background:**

Recent reports suggest that infections due to fluoroquinolone-resistant *Escherichia coli (E. coli)* are an increasingly common complication of transrectal biopsy of the prostate (TBP) in the United States. A better understanding of the magnitude and scope of these infections is needed to guide prevention efforts. Our objective is to determine whether the incidence of infections due to fluoroquinolone-resistant *E. coli* after TBP has increased nationwide in the Veterans Affairs Health Care System and to identify risk factors for infection.

**Methods:**

We performed a retrospective, observational cohort study and a nested case-control study within the US Deparment of Veterans Affairs Healthcare System. The primary outcomes were the incidence of urinary tract infection (UTI) and bacteremia with *E. coli* and with fluoroquinolone-resistant *E. coli* strains within 30 days after TBP. Secondary endpoints focused on the correlation between fluoroquinolone-resistance in all urinary *E. coli* isolates and post-TBP infection and risk factors for infection due to fluoroquinolone-resistant *E. coli* infection.

**Results:**

245,618 patients undergoing 302,168 TBP procedures from 2000 through 2013 were included in the cohort study, and 59,469 patients undergoing TBP from 2011 through 2013 were included in the nested case-control study. Between 2000 and 2013, there was a 5-fold increase in the incidence of *E. coli* UTI (0.18%–0.93%) and a 4-fold increase in the incidence of *E. coli* bacteremia (0.04%–0.18%) after TBP that was attributable to an increase in the incidence of fluoroquinolone-resistant *E. coli* UTI (0.03%–0.75%) and bacteremia (0.01%–0.14%). The increasing incidence of fluoroquinolone-resistant *E. coli* infections after TBP occurred in parallel with increasing rates of fluoroquinolone-resistance in all urinary *E. coli* isolates. By multivariable logistic regression analysis, independent risk factors for fluoroquinolone-resistant *E. coli* UTI after TBP included diabetes mellitus, fluoroquinolone exposure, prior hospitalization, and prior cultures with fluoroquinolone-resistant gram-negative bacilli.

**Conclusion:**

In the Veterans Affairs Healthcare System, the incidence of *E. coli* infection after TBP has increased significantly since 2000 due to a dramatic rise in infections with fluoroquino-lone-resistant *E. coli*.

**STANDFIRST**

In the Veterans Affairs Health Care System, the incidence of *Escherichia coli (E. coli)* infection after transrectal biopsy has increased significantly since 2000 due to a dramatic rise in infections with fluoroquinolone-resistant *E. coli*.

## INTRODUCTION

An estimated 1 million American males undergo transrectal ultrasound-guided biopsy of the prostate (TBP) each year, usually for prostate cancer screening [1]. Because the biopsy needle passes through the rectal mucosa, the TBP procedure is associated with a significant risk of infectious complications, including urinary tract infection (UTI), prostatitis, and bacteremia [2–10]. *Escherichia coli (E. coli)* is the most common cause of these infections. In randomized trials, antibiotic prophylaxis has been shown to significantly reduce the frequency of infectious complications after TBP [11].

Fluoroquinolones are the antibiotics most often used for TBP peri-procedural prophylaxis in the United States [11, 12]. However, fluoroquinolone resistance in *E. coli* and other gram-negative bacilli (GNB) has increased steadily in recent years [13, 14], and there have been several reports of increasing rates of infection due to fluoroquinolone-resistant *E. coli* after TBP, mostly from individual institutions [6–9, 15–19]. In 3 nonrandomized trials, modification of antimicrobial prophylaxis based on pre-procedure rectal culture results has been effective for prevention of infection due to fluoroquinolone-resistant *E. coli* after TBP [20–22]. Modification of prophylaxis regimens for all patients undergoing TBP also has been effective [23–25], but adverse consequences of this approach have been reported [26]. In order to prioritize efforts to develop effective strategies to prevent infections after TBP, there is a need for a better understanding of the magnitude and scope of these infections in the United States.

The Department of Veterans Affairs (VA) operates the largest healthcare system in the United States. *E. coli* is a common cause of infections in veterans, and fluoroquinolone-resistant strains, particularly sequence type 131, are widespread in the healthcare system [27]. Using national VA data for a 14-year period, we tested the hypothesis that the incidence of UTI and bacteremia with *E. coli*, particularly with fluoroquinolone-resistant *E. coli* strains, is increasing after TBP. We conducted a case-control study to identify risk factors for infection due to fluoroquinolone-resistant *E. coli* after TBP. We hypothesized that prior exposure to fluoroquinolones and or previous admission to the hospital would predict most cases of infection with fluoroquinolone-resistant *E. coli* after TBP.

## METHODS

### Protection of Human Research Participants

The institutional review board at the Cleveland VA Medical Center approved all study activities. Informed consent was waived.

### Study Design

Using national VA databases, we determined the incidence of bacteriuria and bacteremia with *E. coli* and of fluoroquinolone-resistant *E. coli* within 30 days after TBP. Rates of post-TBP *E. coli* infections were compared over time and for different geographic regions. We examined the correlation between the percentage of fluoroquinolone-resistance for all urinary and blood *E. coli* and the incidence of post-TBP infection with fluoroquinolone-resistant *E. coli* post-TBP, both overall and by facility. A nested case-control study of patients undergoing TBP from 2011 through 2013 was conducted to identify risk factors for infection with fluoroquinolone-resistant *E. coli*.

### Data Sources and Definitions

We used data initially generated by the VA Electronic Health Record at all 152 VA hospitals and 850 outpatient clinics. The Veterans Informatics and Computing Infrastructure (VINCI) was used to access and analyze data on demographics, laboratory, and microbiology results, comorbidities derived from the Veterans Health Administration Corporate Data Warehouse, and pharmacy data extracted from the Decision Support System, Pharmacy Benefits Management, and the Corporate Data Warehouse. Encrypted patient identifiers were used to linked data from different sources.

We identified patients undergoing TBP in all VA hospitals and outpatient clinics in the VA Health Care System between January 1, 2000 and December 31, 2013 by searching for Current Procedural Terminology (CPT) code 55700, which designates “biopsy, prostate; needle or punch, single or multiple, any approach” and either CPT code 76872 for transrectal ultrasound or 76942 for ultrasonic guidance for the needle biopsy of the prostate. In parallel, we identified the subset of patients undergoing TBP with positive urine or blood cultures for *E. coli* within 30 days after TBP, and determined if the *E. coli* isolates were resistant to fluoroquinolones. We also determined the proportion of all *E. coli* urinary and blood isolates that were resistant to fluoroquinolones during the same time period.

Post-TBP bacteriuria and bacteremia were defined on the basis of a positive culture for *E. coli* within 30 days after TBP in urine or blood, respectively. For the purposes of the study, we classified *E. coli* isolates as fluoroquinolone-resistant if they were “intermediate” or “resistant” based on Clinical and Laboratory Standards Institute criteria for ciprofloxacin or, when not available, levofloxacin. Diabetes was defined based on the documentation of at least one of the appropriate International Classification of Diseases, Ninth Revision (ICD-9) codes at an inpatient VA encounter, or two separate outpatient visits within 12 months prior to the procedure, as described in the Veterans Aging Cohort Study methodology [28]. Prior admission to a VA inpatient facility within the previous year was defined based on documentation of a stay in an inpatient setting in the VA system within 365 days prior to the TBP procedure. Prior fluoroquinolone exposure was defined as a prescription for a fluoroquinolone antibiotic in the 365 days prior to the procedure, excluding the 90 days prior to the procedure because peri-procedural prophylaxis is typically prescribed 1–3 months prior to the procedure.

### Statistical Analysis

The yearly incidence of fluoroquinolone-resistant and fluoroquinolone-susceptible *E. coli* bacteriuria and bacteremia within 30 days after TBP was calculated per 100 procedures. Incidence of infections was compared over time and by VA-defined regions (Eastern, Western, Southern, and Central). For the period 2008 through 2013, we compared the facility-level incidence of post-TBP infection with fluoroquinolone-resistant *E. coli* among facilities with differing prevalence of fluoroquinolone-resistance in urinary *E. coli* isolates (i.e., < 20%; 20%–29.99%; 30%–39.99%; and ≥ 40%).

We conducted a nested case-control study of TBP procedures performed between January 2011 and December 2013. For patients undergoing more than one TBP during this period, we only included the first procedure. Patients who had a bacteriuria with fluoroquinolone-resistant *E. coli* based on a positive urine culture within 30 days after TBP were compared with patients who did not have a urine culture positive for fluoroquinolone-resistant *E. coli*; patients with positive urine cultures for fluoroquinolone-susceptible *E. coli* bacteriuria were excluded from the analysis. Categoric variables were described using proportions and continuous variables were described using median and range. To identify risk factors for post-TBP infection with fluoroquinolone-resistant *E. coli*, we first conducted a univariate analysis to compare the probability of developing a bacteriuria in relation to the following factors: age at the time of the procedure, admission to a VA hospital within the previous year, history of isolation of fluoroquinolone-resistant *E. coli* from a urine culture within the previous year, diabetes mellitus, receipt of a systemic antibiotic within the previous year, and receipt of a fluoroquinolone within the previous year excluding the 90 days prior to the procedure. Fisher's exact test or chi-square test was used to test the strength of association among categoric variables as appropriate. Student's *t*-test was used to compare continuous variables. The statistical threshold for significance was set at *P* = 0.01 for a 2-tailed test. Factors with a *P*-value less than 0.1 on a univariate analysis were placed into a multivariable model to determine the adjusted odds ratios. A *P*-value of 0.1 was selected rather than 0.2 because the small sample limited the number of variables that could be analyzed in a multivariable model. Finally, we calculated the proportion of fluoroquinolone-resistant *E. coli* bacteriuria that would have been detected by combinations of risk factors identified as statistically significant.

## RESULTS

### E. coli Infections After TBP

**Figure 1.**
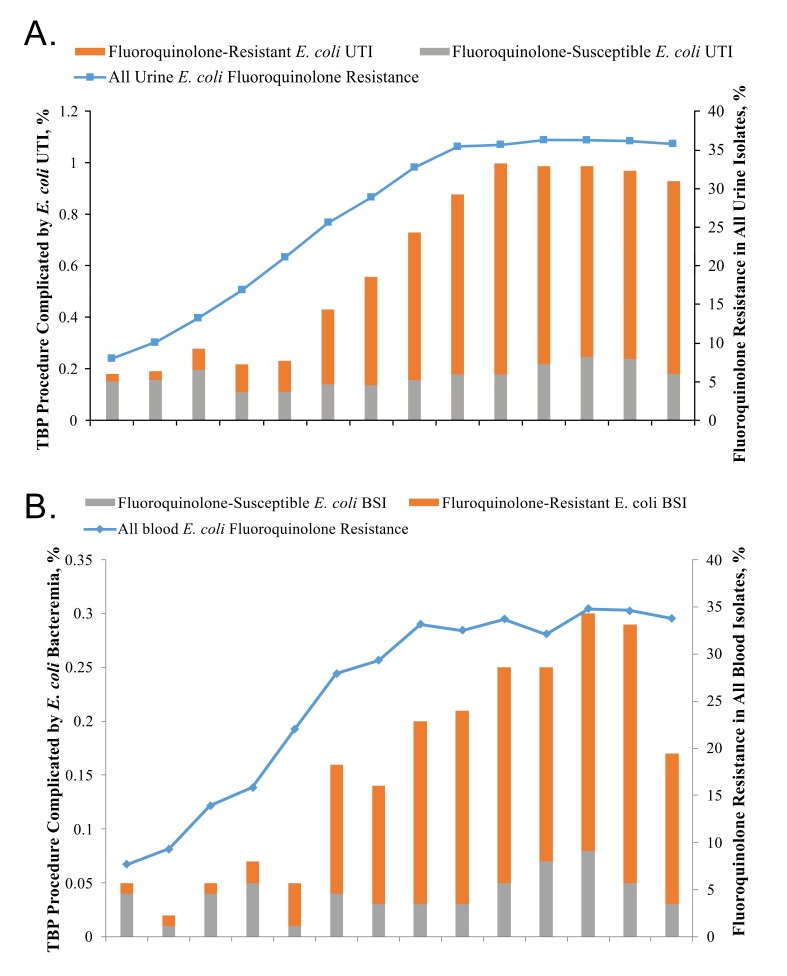
Escherichia coli Infections Following Transrectal Biopsy of the Prostate and Fluoroquinolone Resistance in All E. coli Abbreviations: CI: confidence interval; FQ: fluoroquinolone; OR: odds ratio; TBP: transrectal biopsy of the prostate; UTI: urinary tract infection.

Between January 1, 2000 and December 31, 2013, a total of 302,168 TBP procedures were performed on 245,618 men from 121 VA facilities. The number of TBP procedures performed per facility each year ranged from 1 to 848 (mean, 192; median, 158). Figure 1 shows respectively the incidence of post-TBP bacteriuria and bacteremia due to *E. coli*, stratified by fluoroquino-lone-resistant and fluoroquinolone-susceptible isolates, and the concurrent yearly prevalence of fluoroquinolone resistance in all *E. coli* urinary or blood isolates. Overall, 1,936 (0.64%) TBP procedures were complicated by *E. coli* bacteriuria and 502 (0.17%) were complicated by *E. coli* bacteremia. Between 2000 and 2013, there was a 5-fold increase in the incidence of *E. coli* bacteriuria (0.18% –0.93%; *P* < 0.0001) and a 4-fold increase in the incidence of *E. coli* bacteremia (0.04%– 0.18%; *P* < 0.0001) after TBP. The incidence of post-TBP bacteriuria due to fluoroquinolone-susceptible *E. coli* remained stable during the study period (0.15% and 0.18% in 2000 and 2013, respectively; *P* = 0.5), whereas the incidence of post-TBP bacteriuria due to fluoroquinolone-resistant *E. coli* increased by 25-fold (0.03%–0.75%; *P* < 0.0001), peaking in 2009. The incidence of post-TBP bacteremia due to fluoroquinolone-susceptible *E. coli* remained stable during the study period (0.04% and 0.03% in 2000 and 2013, respectively; *P* = 0.9), whereas the incidence of post-TBP bacteremia due to fluoroquinolone-resistant *E. coli* increased by 23-fold (0.01%–0.14%; *P* < 0.0001), peaking in 2012. During the study, the proportion of post-TBP *E. coli* isolates that were fluoroquinolone-resistant increased significantly for both urinary (16.7%–80.4%; *P* < 0.0001) and bacteremia (14.3%–80.6%; *P* = 0.002) isolates.

The increasing incidence of fluoroquinolone-resistant *E. coli* infections after TBP occurred in parallel with increasing rates of fluoroquinolone-resistance in all urinary and blood *E. coli* isolates (Figure 1). For 671,682 urinary *E. coli* isolates that were subjected to susceptibility testing during the study period, the proportion that was resistant to fluoroquinolones increased from < 10% in 2000 to ~35% in 2008, and subsequently remained stable. Similarly, the proportion of all blood *E. coli* isolates that was resistant to fluoroquinolones increased from less than 10% to ~30% during the study period, peaking in 2011. Of note, results of susceptibility testing to ciprofloxacin and or levofloxacin were available for 97% of samples; when both were available, they were concordant in 99.5% of cases.

**Figure 2.**
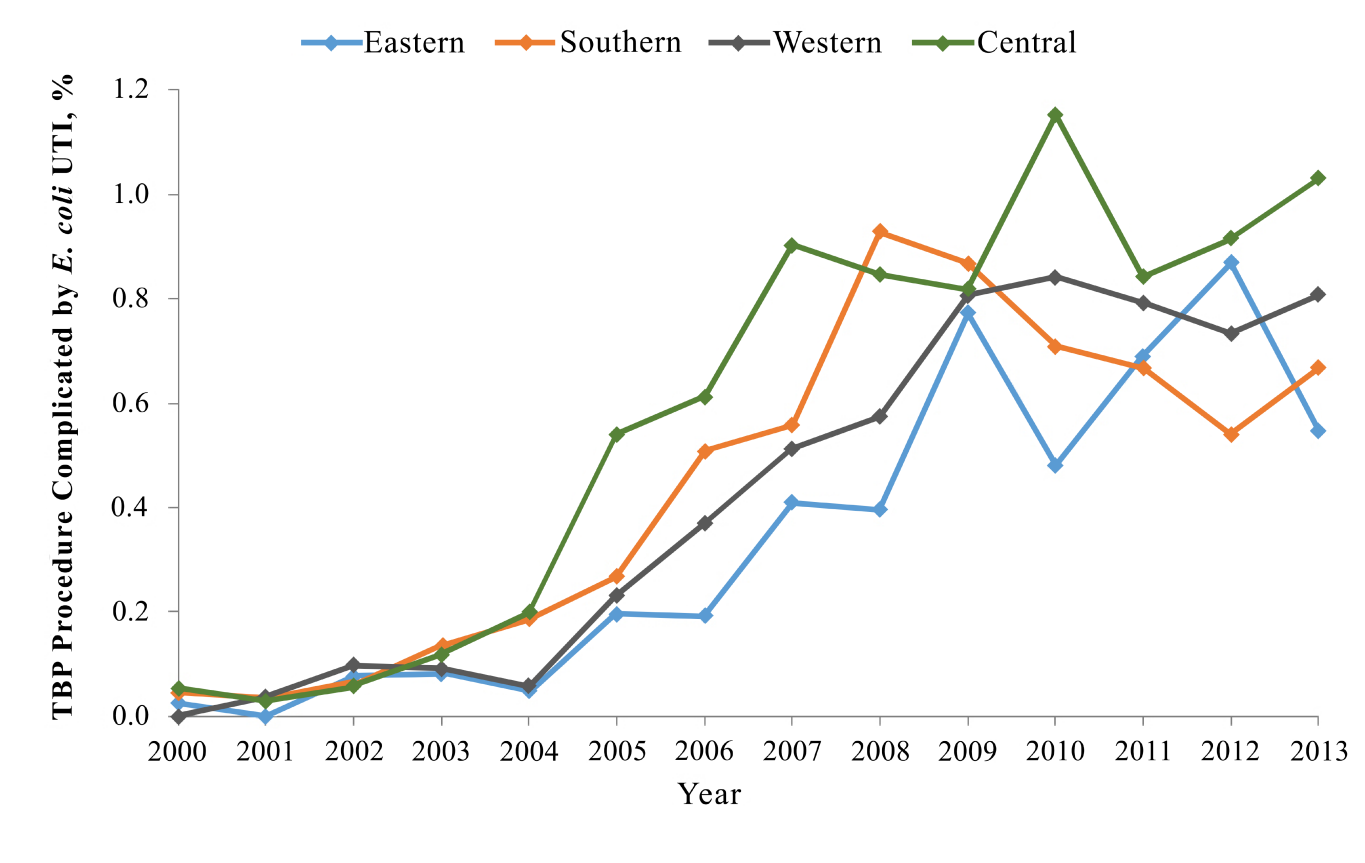
*Escherichia coli* Urinary Tract Infections Following Transrectal Biopsy of the Prostate, by Geographic Region

Figure 2 shows the increase of the incidence of post-TBP fluoroquinolone-resistant *E. coli* bacteriuria during the study period, stratified by region. The increase in the incidence of post-TBP bacteriuria occurred in all regions of the country. The yearly incidence of post-TBP fluoroquino-lone-resistant *E. coli* bacteriuria varied considerably among VA facilities, ranging from 0% to 5.9%.

### Correlation of Prevalence of Fluoroquinolone-Resistance in all *E. coli* Isolates with Incidence of Post-TBP Infection due to Fluoroquinolone-Resistant E. coli by Facility

**Figure 3.**
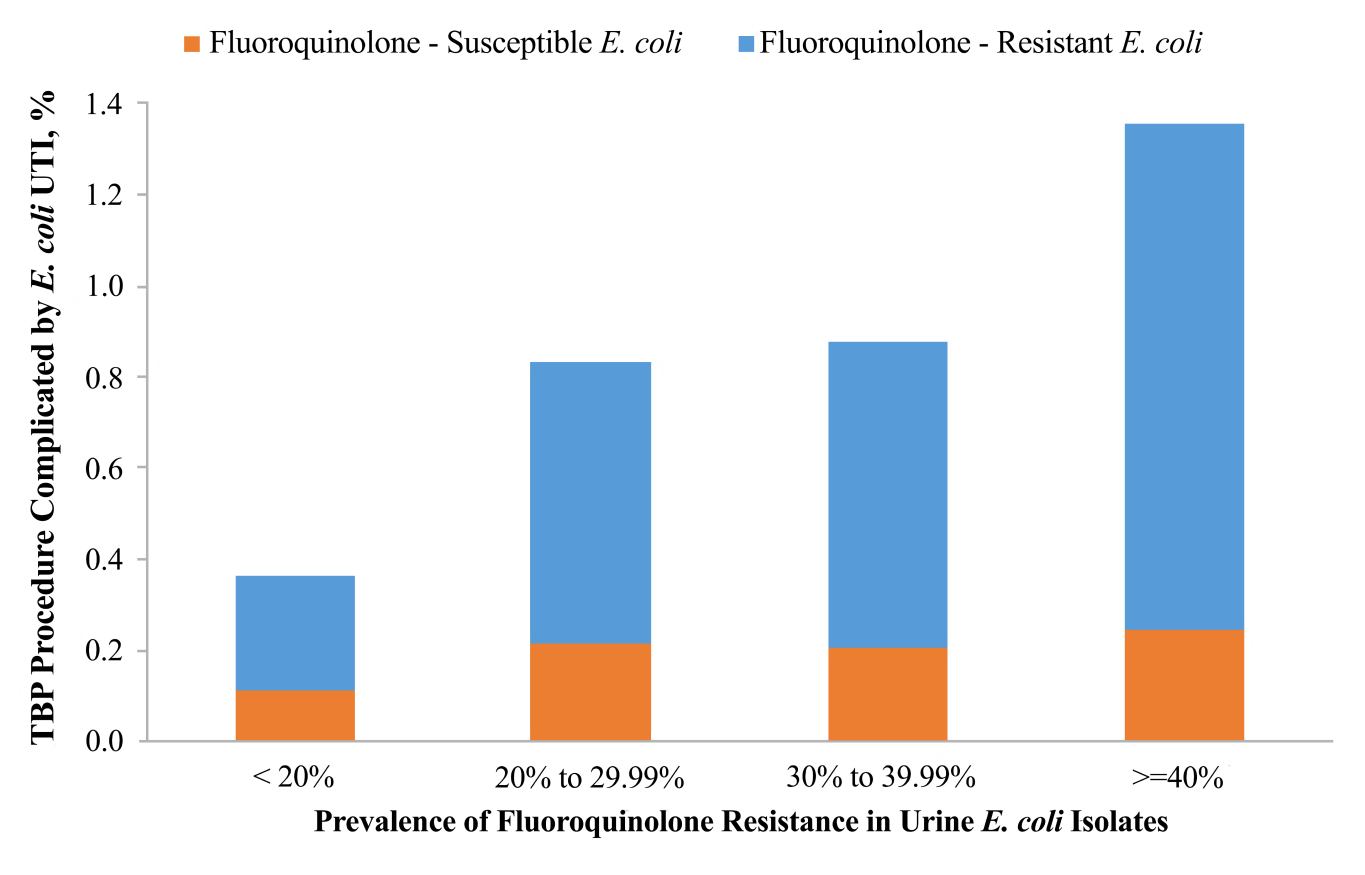
Facility Fluoroquinolone-Resistant *Escherichia coli* Urinary Tract Infections Following Transrectal Biopsy of the Prostate and Fluoroquinolone Resistance in All Urinary *E. coli*

Figure 3 shows the facility-level incidence of post-TBP infection with fluoroquinolone-resistant *E. coli* between 2008 and 2013, stratified by the prevalence of fluoroquinolone-resistance in all urinary *E. coli* isolates. The incidence of post-TBP infection with fluoroquinolone-resistant *E. coli* increased in a stepwise fashion as the prevalence of fluoroquinolone-resistance in all urinary *E. coli* isolates increased. Facilities with < 20% fluoroquinolone-resistance in urinary *E. coli* isolates had a 3-fold lower incidence of post-TBP infection with fluoroquinolone-resistant *E. coli* than facilities with > 40% fluoroquinolone-resistance in urinary *E. coli* isolates.

### Risk Factors for Infection with Fluoroquinolone-Resistant E. coli After TBP

Between 2011 and 2013, a total of 59,613 patients underwent 67,183 TBP procedures. Table 1 shows a comparison of the characteristics of the 428 patients who developed post-TBP bacteriuria with fluoroquinolone-resistant *E. coli* and the 59,041 who did not develop any post-TPB bacteriuria infection with *E. coli* and odds ratios for development of infection. On univariable analysis, hospital admission in the previous year, isolation of a fluoroquinolone-resistant gram-negative bacillus in the previous year, diabetes mellitus, fluoroquinolone exposure in the previous year, and exposure to nonfluoroquinolone antibiotics in the previous year were significantly associated with post-TBP bacteriuria with fluoroquinolone-resistant *E. coli*. In the multivariable model, previous hospital admission, diabetes mellitus, previous isolation of a fluoroquinolone-resistant gram-negative bacillus, and previous exposure to a fluoroquinolone remained significant as independent predictors of infection.

**Table 1.** Risk Factors for Fluoroquinolone-Resistant *Escherichia coli* Bacteriuria After Transrectal Biopsy of the Prostate

Variable	Control (N = 59,041)	FQ-Resistant *E. coli* (N = 428)	Univariable analysis		Multivariable analysis	
			OR (95% CI)	*P* Value	OR (95% CI)	*P* Value
**Age, median (range)**	**65.0 (44-93)**	**64.9 (25–97)**		**0.68**		
**Diabetes mellitus**	**16,940 (28.7)**	**168 (39.3)**	**1.6 (1.3–2.0)**	**< 0.0001**	**1.5 (1.2–1.8)**	**< 0.0001**
**History of a culture positive for FQ-resistant *E. coli***	**283 (0.5)**	**21 (4.9)**	**10.7 (6.8–16.9)**	**< 0.0001**	**7.4 (4.6–11.8)**	**< 0.0001**
**Admission in past year**	**5,013 (8.5)**	**67 (15.7)**	**2.0 (1.5–2.6)**	**< 0.0001**	**1.5 (1.2–2.0)**	**0.003**
**Fluoroquinolone use in the past year**	**11,537 (19.7)**	**122 (27.6)**	**1.6 (1.3–2.0)**	**< 0.0001**	**1.4 (1.1–1.7)**	**0.003**
**Other antibiotic use in the past year^a^**	**16,883 (28.6)**	**153 (35.8)**	**1.4 (1.1–1.7)**	**0.001**	**1.1 (0.90–1.4)**	**0.27**

Abbreviations: CI: confidence interval; FQ: fluoroquinolone; OR: odds ratio.

Note: Data are number (%) of patients unless otherwise specified.

^a^ Included all identified systemic formulations of penicillins, cephalosporins, macrolides, trimethoprim-sulfamethoxazole, tetracyclines, and aminoglycosides.

Although fluoroquinolone exposure was a significant risk factor for post-TBP bacteriuria with fluoroquinolone-resistant *E. coli*, only 27.6% of infected patients had received a fluoroquinolone in the year prior to the biopsy, excluding the 90 days prior to the procedure. In addition, only 15.7% of those with post-TBP bacteriuria with fluoroquinolone-resistant *E. coli* had been admitted to the hospital in the year before the procedure. Moreover, 169 (39.5%) of the 428 patients who developed post-TBP bacteriuria with fluoroquinolone-resistant *E. coli* did not have any of the 4 characteristics identified as predictors of infection by the multivariable model.

## DISCUSSION

In this national cohort study of veterans undergoing TBP, we found that there was a 5-fold increase in the incidence of post-procedure *E. coli* bacteriuria and bacteremia between 2000 and 2013. The increase occurred in all regions of the country and was attributable to a dramatic increase in infections due to fluoroquinolone-resistant *E. coli* strains. The increasing incidence of fluoroquinolone-resistant *E. coli* infections after TBP occurred in parallel with increasing rates of fluoroquinolone-resistance in all urine and blood *E. coli* isolates. These findings build upon previous reports of increasing rates of post-TBP infections in individual hospitals or in multiple hospitals in a locality and suggest that there is an urgent need for development of effective strategies to prevent post-TBP infections.

Our findings suggest that the burden of post-TBP infections is significant in the United States. In the VA population, 0.9% of all TBP procedures in 2013 were complicated by *E. coli* bacteriuria and 0.2% by bacteremia; approximately 80% of these infections are due to fluoroquinolone-resistant *E. coli* isolates. If these frequencies are applicable to the approximately 1 million TBP procedures performed in the general United States population each year, an estimated 9000 *E. coli* UTIs and 2000 *E. coli* bloodstream infections occur yearly as a complication of TBP; more than 7200 of the UTIs and 1600 of the bloodstream infections are due to fluoroquinolone-resistant *E. coli* isolates. These infections may be associated with significant morbidity, costs, and frequent need for hospital admission [15–19]. For example, in a recent report, 51% of patients with post-TBP infection due to fluoroquinolone-resistant gram-negative bacilli required hospital admission, 20% had bacteremia, and 3% had abscess of the prostate [18].

Our findings have several important clinical implications. First, we identified several independent risk factors for fluoroquinolone-resistant *E. coli* bacteriuria after TBP that could be useful to identify a subset of patients at high risk for infection. These included diabetes mellitus, fluoroquino-lone exposure, prior hospitalization, and prior cultures with fluoroquinolone-resistant gram-negative bacilli. However, it is notable that only 27.6% of those with fluoroquinolone-resistant *E. coli* infections had previous fluoroquinolone exposure. Moreover, 39.5% of patients who developed post-TBP bacteriuria with fluoroquinolone-resistant *E. coli* did not have any of the 4 characteristics identified as independent predictors of infection. Second, the incidence of post-TBP infection with fluoroquinolone-resistant *E. coli* increased in a stepwise fashion as the prevalence of fluoroquinolone-resistance in all urinary *E. coli* isolates increased. Thus, monitoring of the prevalence of fluoroquinolone resistance in urinary *E. coli* isolates may provide a useful means to predict risk for post-TBP infections in a facility. Finally, many infection control programs do not conduct routine surveillance for infectious complications after outpatient biopsy procedures because the risk of infection is perceived to be low. Given the increasing prevalence of fluoroquinolone resistance in *E. coli* throughout the United States, routine surveillance for infections after TBP is indicated, particularly when rates of fluoroquinolone resistance are high in urinary *E. coli* isolates.

One notable observation from our study is that the rise in the incidence of *E. coli* infection after TBP peaked in 2008–2012, and has subsequently plateaued. One potential explanation for this observation might be that many urologists may have begun to implement interventions during this time period in response to rising rates of infection. As noted previously, modification of antimicrobial prophylaxis based on pre-procedure rectal culture results and modification of prophylaxis for all patients undergoing TBP have implemented in response to outbreaks, and have been associated with reductions in infection due to fluoroquinolone-resistant *E. coli* after TBP [20–25]. Future studies are needed to determine how widely disseminated such interventions have become. However, although changing strategies for prophylaxis may contribute to the recent reductions in post-TBP infection, the percentage of all *E. coli* urinary and blood isolates that are resistant to fluoroquinolones also has plateaued in the VA system. Thus, other factors may be contributing to the plateau in the incidence of infection after TBP.

There were a number of limitations in this study. First, the study was conducted in the VA health-care system and may not be generalizable to to US population at large. Second, it is possible that we underestimated the number of infections post-TBP because some patients may have sought care for post-TBP infections outside the VA system. Similarly, it is possible we underestimated antibiotic exposures because prescriptions outside the VA system were not captured. Third, we used bacteriuria as a surrogate for urinary tract infection, which would overestimate the incidence of *E coli* infection; additionally, it is not known if bacteriuria with fluoroquinolone-resitant versus susceptible *E coli* leads to different rates of symptomtic urinary tract infection. However, similar trends were observed for bacteremia isolates. Fourth, because fluoroquinolones for TBP prophylaxis are often prescribed during office visits in advance of the procedure, we excluded fluoroquinolone prescriptions in the 90 days prior to the procedure for the analysis of prior fluoroquinolone exposure. Thus, it is possible that we underestimated fluoroquinolone exposures if these agents were prescribed for other reasons in the 90 days before the procedure. Fifth, it is possible that we did not capture all TBP procedures if the procedure codes for transrectal ultrasound or ultrasonic guidance for the needle biopsy of the prostate were not coded for patients undergoing biopsy of the prostate. The transrectal approach is the predominant approach for prostate biopsy, and it has been demonstrated that use of procedural codes indicating ultrasound guidance may lead to decreased capture of qualifying procedures given the underutilization of these codes. Additionally, since medical record review for individual patients was not feasible, our analysis did not include or adjust for potentially important confounders such as case mix, duration of antibiotic exposure, and urologic abnormalities that might increase the risk for infection. Finally, our case-control approach is considered exploratory; a more refined analysis would include additional variables of interest, such as other comorbidities besides diabetes mellitus, as well as a more discerning antibiotic exposure review, and a comparison to similar susceptible infections.

## CONCLUSIONS

In this national cohort of veterans undergoing TBP from 2000 through 2013, there was a 5-fold increase in post-procedure *E. coli* bacteriuria and a 4-fold increase in post-procedure *E. coli* bacteremia that was attributable to a dramatic increase in infections due to fluoroquinolone-resistant *E. coli* and that occurred in parallel with increasing rates of fluoroquinolone-resistance in all urine and blood *E. coli* isolates. Our findings suggest that routine surveillance for postprocedure infections is indicated in all healthcare facilties performing TBP. In addition, studies are needed to identify effective strategies to prevent post-TBP infections.
